# The Salt-Stress Response of the Transgenic Plum Line J8-1 and Its Interaction with the Salicylic Acid Biosynthetic Pathway from Mandelonitrile

**DOI:** 10.3390/ijms19113519

**Published:** 2018-11-08

**Authors:** Agustina Bernal-Vicente, Daniel Cantabella, Cesar Petri, José Antonio Hernández, Pedro Diaz-Vivancos

**Affiliations:** 1 Biotechnology of Fruit Trees Group, Department Plant Breeding, CEBAS-CSIC, Campus Universitario de Espinardo, 25, 30100 Murcia, Spain; tina.cartagena@hotmail.com (A.B.-V.); daniel.cantabella@irta.cat (D.C.); pdv1@um.es (P.D.-V.); 2 IRTA, XaRTA-Postharvest, Edifici Fruitcentre, Parc Científic i Tecnològic Agroalimentari de Lleida, 25003 Lleida, Catalonia, Spain; 3 Departamento de Producción Vegetal, Universidad Politécnica de Cartagena, Paseo Alfonso XIII, 48, 30203 Cartagena, Spain; cesar.petri@upct.es; 4 Department of Plant Biology, Faculty of Biology, University of Murcia, Campus de Espinardo, E-30100 Murcia, Spain

**Keywords:** chlorophyll fluorescence, J8-1 plum line, mandelonitrile, *Prunus domestica*, redox signalling, salicylic acid, salt-stress, soluble nutrients

## Abstract

Salinity is considered as one of the most important abiotic challenges that affect crop productivity. Plant hormones, including salicylic acid (SA), are key factors in the defence signalling output triggered during plant responses against environmental stresses. We have previously reported in peach a new SA biosynthetic pathway from mandelonitrile (MD), the molecule at the hub of the cyanogenic glucoside turnover in *Prunus* sp. In this work, we have studied whether this new SA biosynthetic pathway is also present in plum and the possible role this pathway plays in plant plasticity under salinity, focusing on the transgenic plum line J8-1, which displays stress tolerance via an enhanced antioxidant capacity. The SA biosynthesis from MD in non-transgenic and J8-1 micropropagated plum shoots was studied by metabolomics. Then the response of J8-1 to salt stress in presence of MD or Phe (MD precursor) was assayed by measuring: chlorophyll content and fluorescence parameters, stress related hormones, levels of non-enzymatic antioxidants, the expression of two genes coding redox-related proteins, and the content of soluble nutrients. The results from in vitro assays suggest that the SA synthesis from the MD pathway demonstrated in peach is not clearly present in plum, at least under the tested conditions. Nevertheless, in J8-1 NaCl-stressed seedlings, an increase in SA was recorded as a result of the MD treatment, suggesting that MD could be involved in the SA biosynthesis under NaCl stress conditions in plum plants. We have also shown that the plum line J8-1 was tolerant to NaCl under greenhouse conditions, and this response was quite similar in MD-treated plants. Nevertheless, the MD treatment produced an increase in SA, jasmonic acid (JA) and reduced ascorbate (ASC) contents, as well as in the coefficient of non-photochemical quenching (qN) and the gene expression of *Non-Expressor of Pathogenesis-Related 1* (*NPR1*) and *thioredoxin H* (*TrxH*) under salinity conditions. This response suggested a crosstalk between different signalling pathways (NPR1/Trx and SA/JA) leading to salinity tolerance in the transgenic plum line J8-1.

## 1. Introduction

Salinity or salt stress significantly affects crop productivity, and it is considered as one of the most important abiotic challenges that plant scientists must confront today. Due to the use of saline waters for irrigation, the percentage of land affected by salinity is continuously growing worldwide. When plants are submitted to salt stress conditions, physiological, biochemical, and nutritional disorders occur, limiting plant growth and development and, ultimately, productivity. These deleterious effects are due to the accumulation of toxic ions (Na^+^ and Cl^−^), leading to especially Ca^2+^ and K^+^ deficiency among other nutrient imbalances, and the reduced water uptake produced by osmotic stress [1,2]. In addition, salinity also induced an oxidative stress mediated by reactive oxygen species (ROS) at the subcellular level [2].

Furthermore, it is well known that plant hormones are key factors in the defence signalling output triggered during both abiotic and biotic environmental stress conditions. Among these hormones, SA has attracted much attention, although other plant hormones, such as abscisic acid (ABA) and jasmonic acid (JA), have also been suggested as modulators of plant defence responses.

Considering the important roles of SA during plant responses against stress conditions, SA is of potential agro-economic interest as a modulator of plant plasticity. Although the regulation of SA biosynthesis and the SA-mediated stress tolerance mechanism have not been fully characterised [3], researchers have found that the exogenous application of SA or analogues induce tolerance to several stress conditions [4]. In the same line, we have previously reported in peach (*P. persica* L.) plants that mandelonitrile (MD) is also involved in SA biosynthesis and improves plant performance under biotic and abiotic stress conditions [5]. In *Prunus*, MD is at the hub of cyanogenic glycoside (CNglcs) synthesis and turnover [6]. CNglcs are specialized secondary metabolites that have been linked to plant plasticity improvement against environmental stress conditions. However, CNglcs turnover is highly species dependent [6]. As a result, further studies must be performed to elucidate whether this new SA biosynthetic pathway from MD is also present in other *Prunus* species, and to determine its possible role in plant plasticity under stress conditions. Accordingly, other authors have suggested that SA biosynthesis varies depending on different factors, including the plant species and the environmental conditions [7,8,9].

One common consequence of exposure to stress conditions is the establishment of oxidative signalling that triggers transduction cascades controlling plant development and defence [10]. The major low-molecular-weight antioxidants ascorbate (ASC) and glutathione (GSH) determine the specificity of this oxidative signalling. Thus, ASC and GSH have been shown to be multifunctional metabolites that are important in redox homeostasis and signalling as well as in developmental and defence reactions [11]. The *NON-EXPRESSOR OF PR-PROTEINS1* (*NPR1*) transcription factor, which is activated by SA, is one of the few known redox-regulated signalling proteins in plants, highlighting the crosstalk between the antioxidant metabolism and plant hormones during environmental stress responses. On the other hand, the roles of thioredoxins (Trx) in redox signalling as regulators of scavenging mechanisms and as components of signalling pathways are well established [12]. It has been suggested that SA signalling activates Trx-h5, leading to NPR1 reduction and releasing active monomers that are translocated from the cytosol into the nucleus; this, in turn, activates the expression of defence genes [13,14].

In the present manuscript, we have analysed whether the SA biosynthetic pathway from MD, previously observed in peach [5], is also present in plum *(P. domestica* cv. *Claudia verde*) plants in the presence or absence of NaCl. Moreover, in order to gain deeper knowledge of the SA-mediated defence network in *Prunus*, we have used transgenic plum plants over-expressing four copies of the cytosolic ascorbate peroxidase gene. These transgenic plants with enhanced antioxidant capacity, named line J8-1, have shown higher regeneration efficiency and enhanced vigour as well as tolerance to salt stress under in vitro conditions [15,16]. Moreover, line J8-1 has displayed enhanced tolerance to water stress under greenhouse conditions [17]. Thus, line J8-1 could be an excellent model to study the crosstalk among stress tolerance, oxidative stress and SA in plum plants. Taking into account all the mentioned above, we analysed the effect of MD and Phe (MD precursor) treatments on plant performance (chlorophyll content, chlorophyll fluorescence parameters and leaf and root water contents), on the content of stress-related hormones, on the redox state and the expression of two redox-related genes, and on the soluble leaf and root nutrient content in the transgenic J8-1 line under control and salt stress conditions.

## 2. Results

The following experiments were designed in order to elucidate whether MD could be a precursor of SA biosynthesis in plum plants, as occurred in peach [5]. Moreover, to further study the crosstalk among stress tolerance, oxidative stress, and SA under salinity conditions, the effect of MD and Phe (MD precursor) has been investigated in the transgenic plum line J8-1 submitted to NaCl.

### 2.1. Metabolomic Analysis of SA Biosynthesis in Plum Plants

We have previously described in peach that the cyanogenic glycoside (CNglcs) pathway is involved in SA biosynthesis, suggesting the existence of a third SA biosynthetic pathway, being MD the intermediary molecule between both pathways [5]. Taking into account the fact that the CNglcs pathway is highly dependent on the plant species [6], here we have studied whether this SA biosynthetic pathway, from MD, is also functional in plum plants under control and salinity conditions.

**Figure 1:**
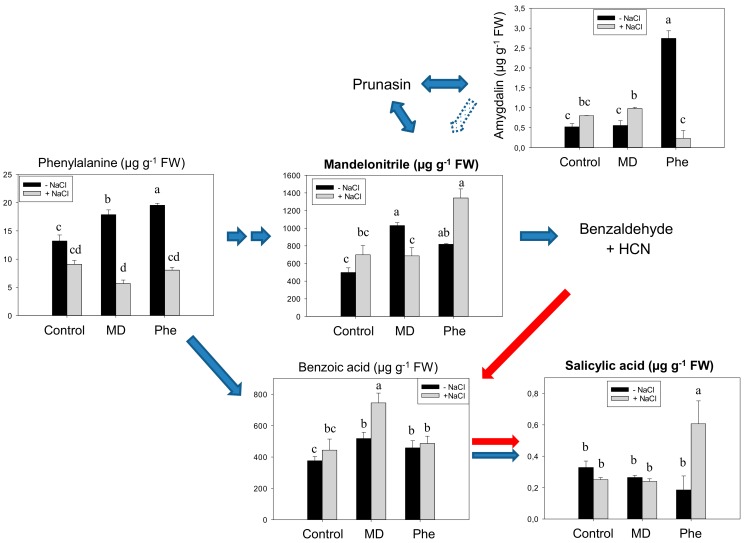
Salicylic acid (SA) biosynthetic and cyanogenic glucoside (CNglcs) pathways in salt-stressed (100 mM NaCl) plum cv. *Claudia verde* shoots micropropagated in the presence or absence of [^13^C]MD or [^13^C]Phe. Total levels (µM g^−1^ FW) of amygdalin, benzoic acid, mandelonitrile, phenylalanine, and salicylic acid are shown. Data represent the mean ± SE of at least 12 repetitions of each treatment. Different letters indicate significant differences in each graph according to Duncan’s test (*p* ≤ 0.05). Blue arrows indicate the previously described SA biosynthesis in higher plants [3] (dot arrow, putative), whereas red arrows show the recently described pathway [5].

When micropropagated non-transgenic plum (cv. *Claudia verde*) shoots were fed with [^13^C]Phe or with [^13^C]MD, in the absence of NaCl, increased levels of Phe, MD and benzoic acid were recorded, whereas amygdalin only increased in Phe-treated shoots (Figure 1). However, contrary to that which we observed in peach [5], none of the treatments produced a significant rise in SA content (Figure 1). In the presence of NaCl, benzoic acid (BA) content only increased in MD-treated micropropagated shoots, whereas only the Phe treatment produced an accumulation of MD and SA (Figure 1).

**Figure 2:**
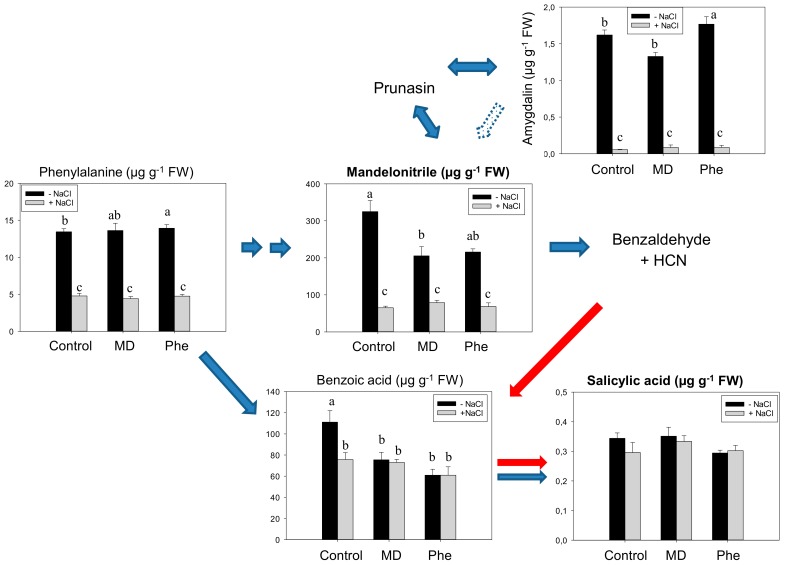
Salicylic acid (SA) biosynthetic and cyanogenic glucoside (CNglcs) pathways in salt-stressed (100 mM NaCl) transgenic J8-1 plum shoots micropropagated in the presence or absence of [^13^C]MD or [^13^C]Phe. Total levels (µM g^−1^ FW) of amygdalin, benzoic acid, mandelonitrile, phenylalanine, and salicylic acid are shown. Data represent the mean ± SE of at least 12 repetitions of each treatment. Different letters indicate significant differences in each graph according to Duncan’s test (*p* ≤ 0.05). Blue arrows indicate the previously described SA biosynthesis in higher plants [3] (dot arrow, putative), whereas red arrows show the recently described pathway [5].

The SA biosynthesis from the CNglcs pathway was also studied in micropropagated shoots from the transgenic plum line J8-1. In the absence of stress, the [^13^C]MD treatment decreased MD and BA levels, while [^13^C]Phe-fed micropropagated J8-1 shoots displayed increased amounts of Phe and amygdalin and lesser amounts of BA (Figure 2). Similar to results in cv. *Claudia verde* and contrary to that which occurred in peach plants [5], neither [^13^C]MD nor [^13^C]Phe increased the SA content under in vitro conditions. Salt stress induced a significant decrease in Phe, MD, and amygdalin in both control and treated (MD or Phe) J8-1 shoots. However, both treatments ameliorated the decrease in benzoic acid observed in control shoots (Figure 2). Regarding SA levels, no statistically significant differences were observed in NaCl-submitted shoots (Figure 2).

Under our experimental conditions, we were able to detect [^13^C]-Phe, -MD and -SA, but no [^13^C]-benzoic acid was observed in either plum plant, cv. *Claudia verde* or the J8-1 line. Regarding the percentage of [^13^C]-labelled compounds, similar values were recorded in both plum plants, and no significant differences were observed among the different treatments and conditions. We observed less than 10% of [^13^C]Phe, and [^13^C]MD but [^13^C]SA values ranged between 20% and 25% of the total amount detected (Supplemental Figure S1). It is noteworthy to mention that, although differences were not statistically significant, the highest levels of [^13^C]MD and [^13^C]SA were observed in [^13^C]MD-fed micropropagated shoots. Moreover, under salinity conditions, no [^13^C]Phe was detected, probably because its rapid turnover under stress conditions (Supplemental Figure S1).

These results suggest that the SA synthesis from the MD pathway demonstrated in peach is not clearly present in plum, at least under in vitro conditions. For this reason, further experiments in order to investigate the crosstalk among stress tolerance, oxidative stress and SA under salinity were performed on the transgenic line J8-1, displaying an enhanced antioxidant capacity.

### 2.2. Effect on Stress-Related Hormones: SA, ABA and JA

It is known that cross-talk among different hormonal signals is involved in different physiological responses as well as in response to environmental challenges. In that sense, the content of SA and other well-known stress-related hormones like ABA and JA was determined in leaves from J8-1 seedlings.

**Figure 3:**
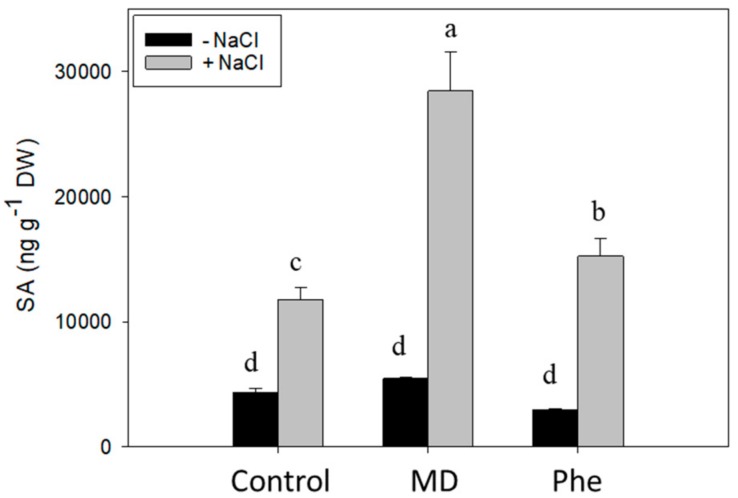
Total SA level (ng g-1 DW) in the leaves of J8-1 seedlings grown in the presence or absence of MD or Phe and submitted to salt stress (6 g/L NaCl). Data represent the mean ± SE of at least four repetitions of each treatment. Different letters indicate significant differences according to Duncan’s test (*p* ≤ 0.05).

In the absence of NaCl, similar to that observed in micropropagated shoots, neither MD nor Phe affected SA levels (Figure 3). Under NaCl stress, however, a significant increase in SA concentration was observed, especially in the presence of MD. In fact, MD-treated J8-1 seedlings showed a 2.3- and 1.7-fold SA increase compared to control and Phe-treated plants, respectively (Figure 3).

**Figure 4:**
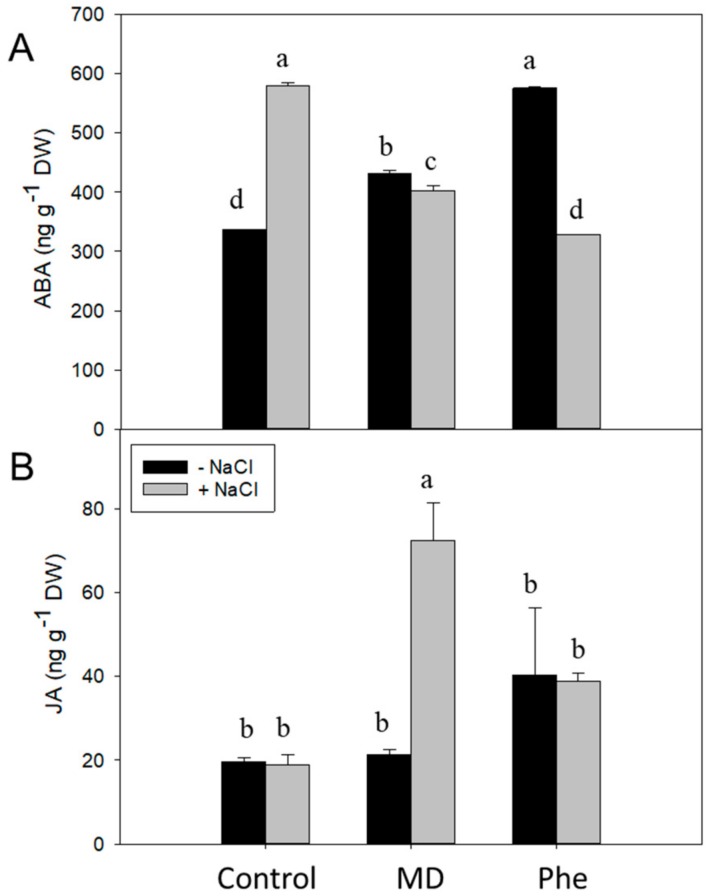
Effect on the stress-related hormones ABA and JA. Total ABA (**A**) and JA (**B**) levels (ng g^−1^ DW) in the leaves of J8-1 seedlings grown in the presence or absence of MD or Phe submitted to salt stress (6 g/L NaCl). Data represent the mean ± SE of at least four repetitions of each treatment. Different letters indicate significant differences according to Duncan’s test (*p* ≤ 0.05).

In addition, we also analysed the levels of other hormones related to stress such as ABA and JA. In the absence of NaCl, both treatments increased ABA levels, with the Phe-treated J8-1 seedlings showing the highest levels (Figure 4A). The presence of NaCl produced a change in this response. In that regards, control plants showed a 1.7-fold increase in ABA levels, whereas in Phe-treated plants, ABA levels declined up to 1.8-fold. Regarding MD-treated J8-1 seedlings, a small but significant decrease in ABA was recorded, in relation to the levels observed in the absence of NaCl (Figure 4A). With regard to the JA concentration, only the MD treatment in the presence of NaCl produced statistically significant changes, increasing considerably the JA levels (Figure 4B).

### 2.3. Plant Growth, Chlorophyll Contents, and Chlorophyll Fluorescence

In previous works, we reported the tolerance of the transgenic plum line J8-1 to salinity (up to 150 mM) under in vitro conditions [16] and to water-stress under ex vitro conditions (up to 15 days of water deprivation) [17]. The NaCl-tolerance was also confirmed under ex vitro conditions as observed by the effect of MD and Phe (MD precursor) treatments on plant performance (chlorophyll content, chlorophyll fluorescence parameters and leaf and root water contents) under salinity stress conditions. Accordingly, NaCl treatment (6 g/L) did not have a significant effect on plant growth (Supplemental Figure S2) or on the leaf water content either in the absence or presence of MD or Phe treatments (Supplemental Figure S3). On the other hand, salinity increased the root water content in control and MD-treated seedlings (Supplemental Figure S3).

**Figure 5:**
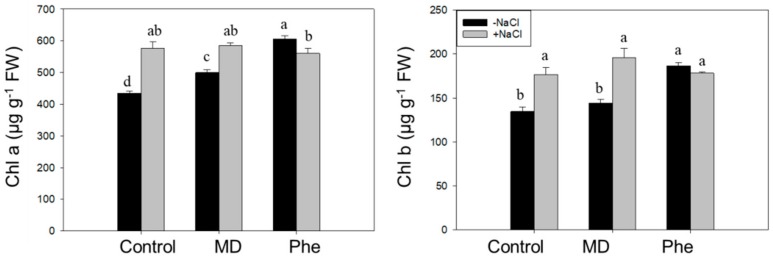
Effect of NaCl (6 g/L) on Chla and Chlb content in control, MD, and Phe treated J8-1 plum seedlings. Data represents the mean ± SE of at least four repetitions. Different letters indicate statistical significance according to Duncan’s test (*p* < 0.05).

We also analysed the effect of NaCl in the presence or absence of MD and Phe treatments on the chlorophyll content in leaves from J8-1 seedlings. In the absence of NaCl, MD treatment increased the Chla content, whereas Phe produced a rise in Chla and Chlb. Under salinity conditions, an increase in Chla and Chlb was observed in non-treated plants as well as in the presence of MD. However, a decrease in Chla was produced in Phe-treated plants (Figure 5).

**Figure 6:**
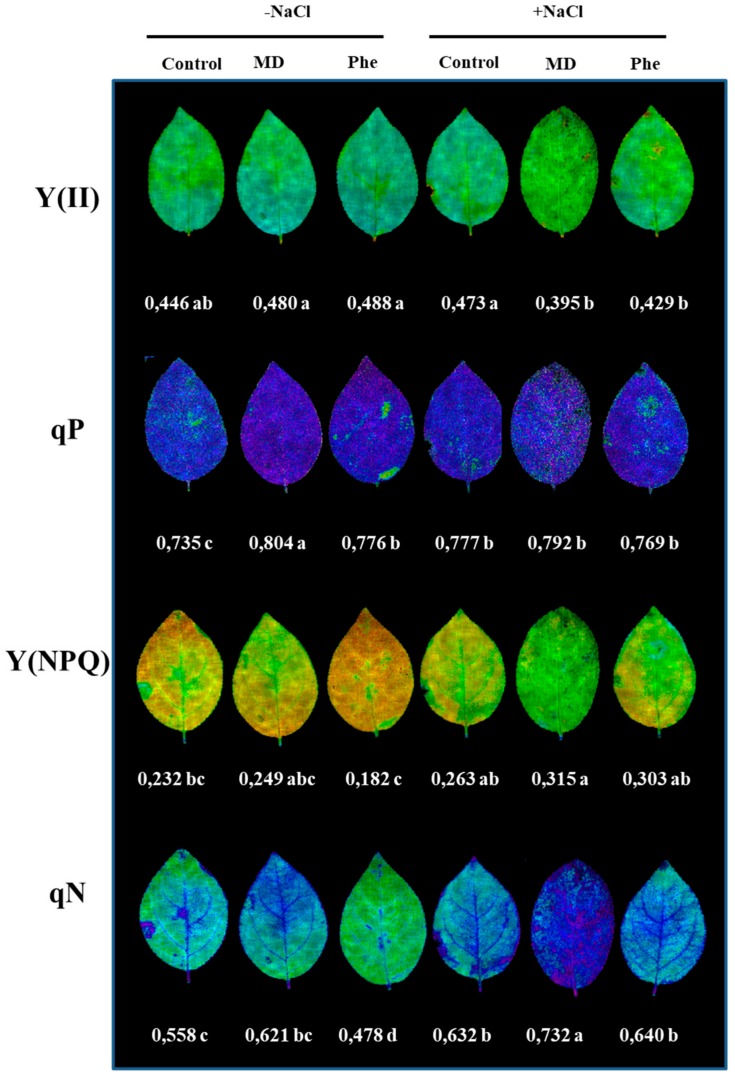
The effect of salt stress (6 g/L NaCl) on the chlorophyll fluorescence parameters in J8-1 seedling leaves. Representative images of the quantum yield of photochemical energy conversion in PS II [Y(II)], the photochemical quenching (qP) and the quantum yield of regulated non-photochemical energy loss in PS II and its coefficient [y(NPQ) and qN] are shown. Zero represents the lowest value and 1 the maximum value for each parameter. The averages of the values of the different parameters analysed are displayed below each image. Data represent the mean ± SE of at least six repetitions. Different letters indicate statistical significance according to Duncan’s test (*p* < 0.05).

In addition to chlorophyll content determination, different photochemical [Y(II) and qP] and non-photochemical [(Y(NPQ) and qN] quenching chlorophyll fluorescence related parameters were also analysed. Under control conditions, both treatments increased qP, whereas a decrease in qN occurred in Phe-treated plants. Under NaCl stress, non-treated plants showed an increase in qP and qN (Figure 6). The MD treatment decreased the photochemical quenching parameters, but an increase in the non-photochemical quenching parameters took place. Regarding the Phe treatment, a decrease in Y(II) was observed, but qP did not show statistically significant changes, whereas, similar to the MD treatment, a significant increase in the non-photochemical quenching parameters occurred (Figure 6).

### 2.4. Redox State and the Gene Expression of Redox-Related Genes

It is well known that the stress hormone SA can interact with the antioxidant metabolism modulating cellular redox homeostasis. For this raison, we determined the redox state in micropropagated J8-1 shoots and in leaves from J8-1 seedlings by analysing the ascorbate and glutathione levels in the absence and in the presence of NaCl.

Under control conditions, micropropagated shoots did not show significant changes in ascorbate or glutathione levels (Table 1 and Table 2). In the presence of NaCl, MD treatment decreased the total (TASC) and reduced ascorbate (ASC) levels, whereas Phe increased the TASC content. As a result, a decrease in the redox state of ascorbate (ASC/TASC) occurred in Phe-treated plants (Table 1). Regarding glutathione levels, an increase in its reduced form (GSH), as well as in the total glutathione (TGSH) level, was only observed in MD-treated micropropagated shoots under salt stress. However, no changes in the redox state of glutathione (GSH/TGSH) were observed in any treatments (Table 2).

The response was rather different in J8-1 seedlings. In this case, under our experimental conditions, we were not able to detect oxidised ascorbate, so only ASC content is shown (Table 3). In absence of NaCl, and similar to that observed under in vitro conditions, no significant differences were apparent in the ASC and GSH levels. When plants were subjected to saline stress, MD treatment increased ASC but decreased GSH. However, MD and Phe treatments induced an accumulation of oxidised glutathione (GSSG), leading to a decrease in the redox state of glutathione in both cases (Table 3).

**Figure 7:**
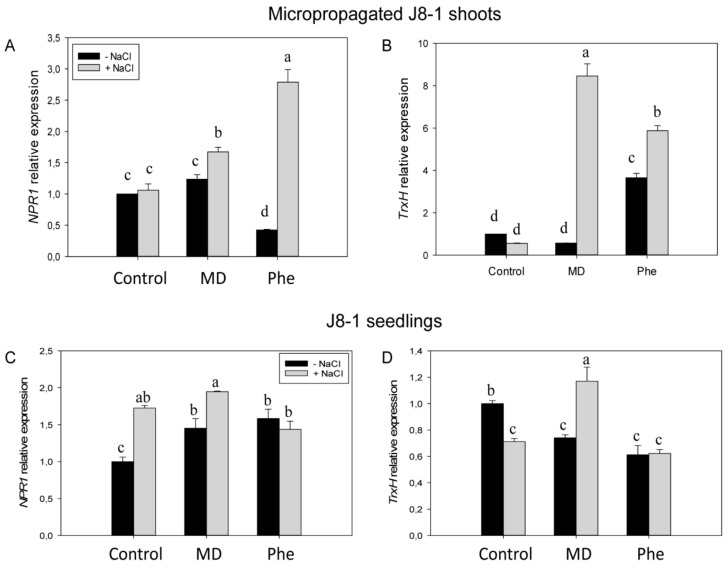
Gene expression of *NPR1* and *TrxH* in micropropagated J8-1 shoots (**A**,**B**) and in the leaves of J8-1 seedlings (**C**,**D**) grown in the presence or absence of MD or Phe and submitted to salt stress. Data represent the mean ± SE of at least five repetitions of each treatment. Different letters indicate significant differences in each graph according to Duncan’s test (*p* ≤ 0.05).

We also studied the effect of MD and Phe treatments on the *Non-Expressor of Pathogenesis-Related 1* (*NPR1*) and *thioredoxin H* (*TrxH*) gene expression levels in NaCl-stressed J8-1 micropropagated plum shoots and in leaves from J8-1 plum seedlings. In the absence of NaCl, micropropagated shoots treated with Phe showed reduced *NPR1* expression but induced *TrxH* expression (Figure 7A,B). In the presence of NaCl, both treatments increased the expression of the studied redox-related genes. The induction was especially striking for the effect of Phe on *NPR1* expression (nearly a five-fold increase) and for the increase in *TrxH* expression (13-fold) observed as a result of MD treatment (Figure 7A,B).

The effect of the treatments on the expression of both redox-related genes in the transgenic plum seedlings was somewhat different. In the absence of NaCl, both MD and Phe treatments induced *NPR1* expression but reduced *TrxH* expression in a similar manner (Figure 7C,D). Under salinity conditions, control and MD-treated seedlings increased *NPR1* expression, whereas *TrxH* expression was repressed in control seedlings but was again induced by MD (Figure 7C,D).

### 2.5. Effect of MD and Phe on Soluble Leaf and Root Nutrient Content under Salt Stress Conditions

**Figure 8:**
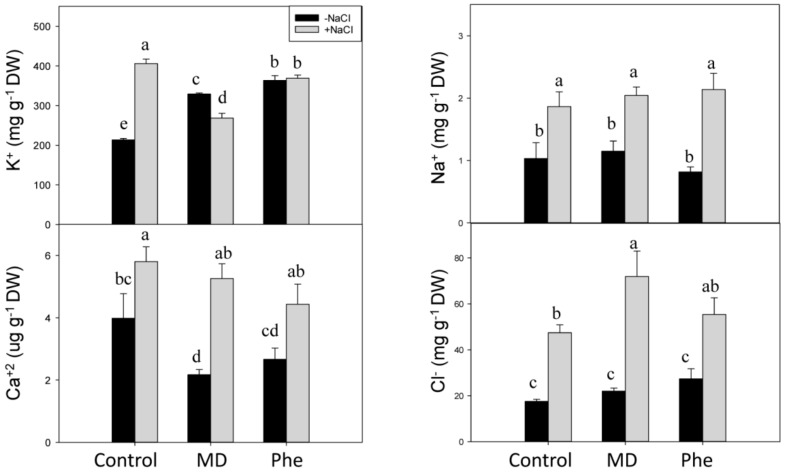
Effect of salt stress (6 g/L NaCl) on soluble K^+^, Ca^2+^, Na^+^, and Cl^−^ contents in leaves from control, MD- and Phe-treated J8-1 seedlings. Data represent the mean ± SE of at least four repetitions. Different letters indicate statistical significance according to Duncan’s test (*p* < 0.05).

Salt stress produced ion toxicity associated with excess Cl^−^ and Na^+^ uptake, leading to Ca^2+^ and K^+^ deficiency and other nutrient imbalances [2]. Therefore, the effect of MD and Phe treatments on soluble K^+^, Ca^2+^, Na^+^ and Cl^−^ levels was analysed in leaves and roots from transgenic plum seedlings grown in the presence and absence of NaCl. Under control conditions, MD and Phe increased leaf K^+^ content but decreased leaf Ca^2+^ content. No effects of MD or Phe treatments on Na^+^ and Cl^−^ levels were observed (Figure 8). In NaCl-stressed seedlings, an increase in all the analysed nutrients occurred in the leaves from non-treated plants. Similar results were observed in the MD and Phe treatments, in which the leaves of salt-stressed seedlings also displayed increased Ca^2+^, Na^+^, and Cl^−^ levels, although the K^+^ level slightly decreased due to MD and was not affected by Phe. It is important to note the low leaf Na^+^ levels found in the transgenic plum seedlings (Figure 8).

**Figure 9:**
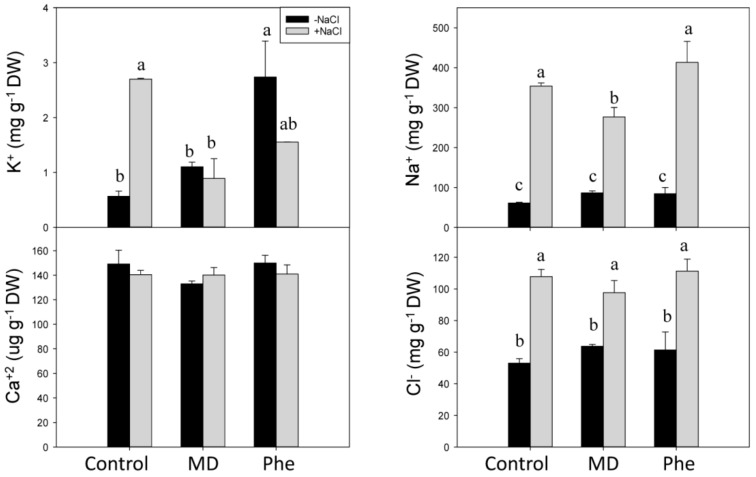
Effect of salt stress (6 g/L NaCl) on soluble K^+^, Ca^2+^, Na^+^, and Cl^−^ contents in roots from control, MD- and Phe-treated J8-1 seedlings. Data represent the mean ± SE of at least four repetitions. Different letters indicate statistical significance according to Duncan’s test (*p* < 0.05).

In the absence of NaCl stress, the only significant change observed in roots was an increase in soluble K^+^ in Phe-treated plants (Figure 9). In the presence of NaCl, an accumulation of the phytotoxic ions Na^+^ and Cl^−^ was observed in control and MD- and Phe-treated seedlings. However, the Na^+^ accumulation in roots was lower in MD-treated plants than in the other treatments. The K^+^ content increased in non-treated NaCl-stressed roots, whereas no significant differences were observed in Ca^2+^ levels in any case (Figure 9).

## 3. Discussion

### 3.1. Involvement of MD on SA Biosynthesis in Plum

In a previous work, we reported that the transgenic plum line J8-1 was tolerant up to 150 mM NaCl under in vitro conditions. This response correlated with high ascorbate peroxidase (APX) activity and gene expression and glutathione and ascorbate contents [16]. We also demonstrated that APX overexpression in line J8-1 can play a major role in the response of J8-1 seedlings to drought conditions by inducing changes at the physiological, biochemical, proteomic, and genetic levels [17]. In our opinion, it is of interest to characterise the response of this transgenic line to NaCl stress under ex vitro conditions. In addition, we recently reported that MD is the intermediary molecule between a suggested new SA biosynthetic pathway and CNglcs turnover in peach plants [5]. All these findings led us to investigate not only the response of the J8-1 line to salinity, but also whether the new SA pathway described in peach plants [5] is also present in this line and the possible role of this pathway on plant performance.

In micropropagated peach shoots fed with [^13^C]MD, nearly 20% of the total SA quantified appeared as [^13^C]SA, demonstrating that MD can be an intermediary molecule in this novel pathway controlling amygdalin and SA biosynthesis [5] However, when micropropagated plum “*Claudia Verde*” shoots were fed with [^13^C]MD or [^13^C]Phe, no increases in SA were detected, although significant increases in MD, Phe and benzoic acid (SA-precursor) were observed. We also assayed this possibility using micropropagated J8-1 shoots. However, the results concerning the involvement of MD as a putative intermediary of SA biosynthesis in this plum line were negative. These results led to the hypothesis that the new SA synthesis pathway from MD, previously demonstrated in peach, seemed not to be operative in plum under in vitro conditions. Nevertheless, we observed that NaCl stress affected the CNglcs pathway, mainly at the MD and amygdalin levels. Due to the fact that amygdalin is derived from MD by the addition of two glucose molecules, it is logical to assume that the amygdalin content decreases. The glucose molecules could be used for osmotic adjustment or to obtain energy for different metabolic processes.

As a conclusion, it seems that MD is not an intermediary for SA biosynthesis in micropropagated plums as it was found to be in micropropagated peach shoots, unless the synthesized SA appears as conjugated form. However, an increase in SA was produced in J8-1 plum seedlings when NaCl stress was imposed, especially in MD-treated plants, suggesting that MD could be involved in the SA biosynthesis in plum grown in the presence of NaCl under greenhouse conditions. These findings are different to the results found in peach, where the contribution of this pathway to the SA pool does not seem to be relevant under salt stress or *Plum pox virus*-infection conditions [18].

### 3.2. Plant Performance of Plum under NaCl Stress

As expected, line J8-1 was also tolerant to high NaCl levels under greenhouse conditions, as observed by the lack of negative effects on plant growth (measured as shoot biomass fresh weight) or in the leaf and root water levels, both in control and MD- and Phe-treated plants. In addition, chlorophyll levels can be seen as a biochemical marker of salt tolerance in plants; the maintenance or the increase in Chl content under NaCl stress can be considered as a protection mechanism for the photosynthesis process. In this regards, both non-treated and MD-treated plants showed increases in Chla and Chlb levels. Moreover, J8-1 plants increased/and or maintained the non-photochemical quenching parameters under NaCl stress, especially in the presence of MD. The maintenance of the non-photochemical quenching parameters under stress situations has been associated with a capacity to dissipate light energy safely, and it can be seen as an adaptive mechanism to protect the chloroplasts under NaCl conditions, avoiding the over-generation of ROS, as described for other plant species [2,19,20,21].

Thus, according to plant growth, leaf and root water content, chlorophyll contents and chlorophyll fluorescence data, the transgenic plum line J8-1 can be considered as salt-tolerant.

### 3.3. Stress-Related Hormones and NaCl Response

The effect of MD and Phe treatments on the stress-related hormones ABA and JA in line J8-1 was quite different to that observed in peach plants. In peach, in the absence of NaCl, the treatments had no effect on ABA and reduced the JA levels. Under saline conditions, MD decreased ABA and JA concentrations, whereas Phe produced a decrease in JA [18]. The transgenic line J8-1 showed contrasting results. In this case, the differences could be due to the use of different plant species showing a different NaCl tolerance and the different NaCl levels used: peach plants were subjected to 2 g/L NaCl, whereas plum plants were treated with 6 g/L NaCl.

Some studies have suggested a positive interaction between SA, JA, ethylene and ABA signalling pathways, improving the response of plants to environmental stresses [22]. JA and SA can regulate plant responses to abiotic stresses. Accordingly, the exogenous application of both JA and SA has been found to enhance salt-tolerance in some plant species by increasing their antioxidative capacity [23,24]. In addition, an increase in the SA/JA ratio has been suggested as a marker of salt stress [25]. In the current study, an increased SA/JA ratio due to salinity was observed in control and MD- and Phe-treated seedlings, the increase being much greater in MD-treated plants. This response was mainly due to the sharp increase in SA levels in MD-treated J8-1 seedlings under salinity conditions. In peach plants, the MD treatment slightly decreased the SA/JA ratio, and no effect of NaCl on plant development was observed [18]. In a study of the salt-tolerant sweet-potato genotype ND98, the JA concentrations in the leaves and roots increased after 12 h of saline treatment (200 mM NaCl), and this response correlated with a regulated stomatal closure [26].

ABA is a well-known regulator of stomatal regulation and, hence, of the abiotic stress response. In the current study, Phe treatment decreased the ABA content, whereas a small decrease occurred in MD-treated plants and an increase was observed in untreated seedlings under salinity conditions. Moreover, SA is also involved in stomatal regulation, and SA treatment has been found to decrease the stomatal aperture in Arabidopsis [27]. Accordingly, the SA increase, and the scanty effect in ABA observed in MD-treated plants in the current study suggests more efficient stomatal regulation under saline conditions.

Considered together, all of this data suggests that MD could have a positive effect on the J8-1 response to salinity through an increase in SA and JA and tight control of the ABA levels.

### 3.4. NaCl Effects on Redox State and Ion Homeostasis

In micropropagated J8-1 shoots, GSH levels increased under salt stress, especially in the MD treatment. Furthermore, this increase correlated with the induction of *NPR1* and *TrxH*, suggesting a role for GSH in the stress-induced expression of these redox-related genes. It seems that GSH could play a role in *NPR1* induction under in vitro conditions. In micropropagated peach shoots, treatment with the artificial precursor of cysteine, L-2-oxothiazolidine-4-carboxylic acid (OTC), produced an increase in GSH and in the GSH/GSSG ratio as well as in the *NPR1* expression both in healthy and in *Plum pox virus*-infected shoots [28]. On the other hand, in J8-1 seedlings, the increase in *NPR1* and *TrxH* expression due to the MD treatment correlated with a decrease in the GSH redox state. As mentioned, under stress conditions, the MD treatment produced a sharp increase in SA content, and researchers have shown that SA-induced changes in glutathione lead to a more oxidised environment that modulates the plant defence responses [29,30,31]. Similarly, MD treatment also leads to a more oxidised environment in peach plants via changes in non-enzymatic and enzymatic antioxidant levels that could be responsible for the modification of the function of redox-regulated proteins such as NPR1 [5]. In absence of stress this MD-induced oxidised environment was due to a decrease in ASC content and GSH redox state in both peach [5] and J8-1 seedlings, whereas in J8-1 seedlings submitted to salinity the decrease in the GSH redox state was accompanied by an increase in ASC. This increase in ASC could be also related to the salt stress tolerance displayed by J8-1 seedlings [4,11,16].

Thioredoxins (Trx) are ubiquitous disulfide reductases that regulate the redox status of target proteins and seem to be involved in the protection of plant cells in stress situations that induce oxidative stress [12]. Trx can prevent the oxidative damage of important macromolecules, thus protecting plants against the stress-induced lipid peroxidation of membranes or repairing oxidised proteins [12]. Proteomic tools have made it possible to identify many potential targets of Trx, including many proteins related with important cellular processes [32]. One of the proteins regulated by thioredoxins is NPR1. Cytosolic Trxs catalyze the redox changes in NPR1 from oligomeric to monomeric forms, with SA inducing TRX-5h to catalyse NPR1 monomer release and to prevent re-oligomerization [13]. Different studies have reported the induction of TrxHs by abiotic or biotic stresses [33], suggesting that these proteins can act as antioxidants in vivo [34].

In the current study, the induction of *TrxH* gene expression by MD was more evident under in vitro conditions, where the absence of the root system leads to more severe symptoms under saline conditions. In plum seedlings (ex vitro conditions), the induction of *TrxH* was lower (only a 1.6-fold increase), which was similar to that observed in the induction of *Trxh1* in rice plants treated with 100 mM NaCl [35]. In addition, the effect of salinity on *TrxH* expression was very similar to that observed in peach [18]: in the absence of chemical treatments, salinity reduced the expression of *TrxH*, whereas induction occurred in the presence of MD, and no changes were produced in the presence of Phe.

Elevated SA levels may mediate adaptive responses against salt stress through NPR1-dependent and NPR1-independent pathways. Salt stress (100 mM NaCl) was found to have a strong effect on plant growth in the Arabidopsis *npr1-5* mutant, which lacks the NPR1-dependent SA signalling pathway. However, the effect of NaCl stress on the plant growth of the Arabidopsis *nudt7* mutant, which constitutively expressed NPR1-dependent and NPR1-independent SA signalling, was more attenuated [36]. In addition, the *npr1-5* mutant was unable to control the Na^+^ influx and prevent K^+^ loss in shoots and roots, in contrast to the results observed in the *nudt7* mutant. These authors concluded that the constitutive expression of NPR1-dependent SA signalling enhanced salt tolerance by controlling Na^+^ entry into roots and shoots as well as minimising K^+^ loss during NaCl challenges, which is an important component of salt and oxidative stress tolerance in Arabidopsis [36]. This information agrees with our results, since under saline conditions, MD-treated plants showed elevated SA levels as well as *NPR1* and *TrxH* expression and less Na^+^ accumulation in roots than the other treatments. This effect of SA on Na^+^ levels has also been observed in pea plants [37]. These authors reported that SA treatment reduced Na^+^ accumulation in pea roots in the presence on 70 mM NaCl.

In the salt-tolerant sweet potato genotype ND98, the JA content increased in the leaves and roots after 12 h of saline treatment (200 mM NaCl), and this response correlated with regulated stomatal closure, reduced Na^+^ accumulation and increased K^+^ concentrations. Furthermore, this genotype showed a more balanced ion homeostasis than the salt-sensitive genotype [26]. In the current study, this response was also partially observed in MD-treated plants under saline stress. In this case, the increase in JA produced by MD treatment in the presence of NaCl correlated with a lower Na^+^ accumulation in roots compared with the other treatments.

In MD- and Phe-treated peach seedlings, an accumulation of saline ions in roots was recorded, suggesting that both treatments could trigger different mechanisms leading to the development of adaptive responses against salinity [38]. These results contrast to those obtained in MD-treated J8-1 seedlings submitted to salinity conditions, which showed a strong increase in leaf soluble Ca^2+^, which correlated with increased SA and JA contents. However, the soluble Ca^2+^ accumulation in leaves was also observed in control and Phe-treated plants that showed increased SA but no change in JA levels. It is important to note that the Ca^2+^ levels observed in leaves from MD- and Phe-treated J8-1 seedlings were lower than in control plants, and a similar response was observed in peach seedlings, suggesting that Ca^2+^ ions could be chelated by organic molecules like MD and Phe [38]. In pea plants, treatment with exogenous SA (50–100 µM) induced an increase of Ca^2+^ in shoots but not in roots, although under NaCl stress (70 mM), the presence of SA did not prevent a NaCl-induced decrease in Ca^2+^ levels [37]. It has been suggested that SA-induced Ca^2+^ contents can also lead to stomatal closure [39]. In J8-1 plants, increases in leaf SA levels under salinity conditions seem to be related to increases in Ca^2+^ in leaves that could lead to tight stomatal control, thus providing protection to membranes under stress conditions.

## 4. Material and Methods

### 4.1. Plant Material

The assays were performed on micropropagated plum [*Prunus domestica* cv. *Claudia verde* and transgenic line J8-1 [16,17]] shoots and J8-1 seedlings, which were submitted to NaCl stress in the presence or absence of MD and Phe (MD precursor) treatments.

The micropropagated plum shoots were subcultured at four-week intervals for micropropagation and samples were taken at the end of the second subculture in the presence of MD and Phe treatments. In the micropropagated shoots, salt stress was imposed by adding 100 mM NaCl to the micropropagation media in the presence or absence of 200 µM [^13^C]MD or [^13^C]Phe (Campro Scientific GmbH, Germany), as described in Diaz-Vivancos et al. (2017) [5]. Seedlings were obtained from rooted and acclimatized to ex vitro conditions J8-1 plantlets. Under greenhouse conditions, J8-1 seedlings were grown in 2 L pots during two months. Then seedlings were submitted to an artificial rest period (eight weeks) in a cold chamber to ensure uniformity and fast growth. After the rest period, seedlings were irrigated once a week with 6 g/L NaCl in the presence or absence of 1 mM MD or Phe for seven weeks. Samples were taken at the end of this period. For all the conditions, 12 seedlings were assayed, and another 12 plants were kept as control.

### 4.2. Metabolomic Analysis

Micropropagated shoots leaf samples (0.5 g FW) were extracted in 50% methanol (1/3 *w*/*v*) and then filtered in PTFE 0.45 µm filters (Agilent Technologies, Palo Alto, CA, USA).The levels of Phe, MD, amygdalin, benzoic acid and SA were determined in micropropagated shoots at the Metabolomics Platform at CEBAS-CSIC (Murcia, Spain) using an Agilent 1290 Infinity UPLC system coupled to a 6550 Accurate-Mass quadrupole TOF mass spectrometer (Agilent Technologies, Palo Alto, CA, USA) [5]. The hormone levels (ABA, JA and SA) in the dry leaves of J8-1 seedlings treated with MD or Phe (0.2 g DW) were determined using a UHPLC-mass spectrometer (Q-Exactive, ThermoFisher Scientific, Barcelona, Spain) at the Plant Hormone Quantification Platform at IBMCP (Valencia, Spain).

### 4.3. Chlorophyll Determination and Chlorophyll Fluorescence

Approximately 0.2 g of the leaves from the J8-1 seedlings submitted to NaCl stress in the presence or absence of MD and Phe were incubated in 50 mL of 80% acetone (*v*/*v*) for 72 h under darkness. The chlorophyll a (Chla) and chlorophyll b (Chlb) content was analysed by measuring the absorbance at 663 and 645 nm [40].

Chlorophyll fluorescence parameters were measured in detached leaves from J8-1 seedlings submitted to NaCl stress in the presence or absence of MD and Phe treatments using a chlorophyll fluorimeter (IMAGIM-PAM M-series, Heinz Walz, Effeltrich, Germany). After a dark incubation period (15 min), the leaves’ minimum and maximum fluorescence yields were monitored. Kinetic analyses were carried out as previously described [21], and the effective PSII quantum yield [Y(II)], the coefficients of photochemical quenching (qP) and non-photochemical quenching (qN), and the quantum yield of regulated energy dissipation [(Y(NPQ)] were recorded.

### 4.4. Ascorbate and Glutathione Analysis

Micropropagated J8-1 line shoot and seedling leaf samples were snap-frozen in liquid nitrogen and stored at −80 °C until use. The frozen samples were homogenised (1/3 *w*/*v*) with 1 M HClO_4_ containing 1 mM polyvinylpolypyrrolidone and 1 mM EDTA. Homogenates were centrifuged at 12,000× *g* for 10 min, and the supernatant was neutralised with 5 M K_2_CO_3_ to pH 5.5–6. The homogenate was centrifuged at 12,000× *g* for 1 min to remove KClO_4_. The supernatant obtained was used for ascorbate and glutathione determination as previously described [41,42].

### 4.5. Gene Expression

We studied the expression levels of the redox-regulated genes *NPR1* (*Non-Expressor of Pathogenesis-Related Gene 1*) and *TrxH* (*thioredoxin H*). Briefly, micropropagated shoots and leaf samples from line J8-1 were snap-frozen in liquid nitrogen and stored at −80 °C until use. RNA was extracted using the Power Plant RNA Isolation kit (Mo Bio), according to the manufacturer’s instructions. The primer sequences were as follows: *NPR1* (forward 5′-tgcacgagctcctttagtca-′3; reverse 5′-cggcttactgcgatcctaag-′3); *TrxH* (forward 5′-tggcggagttggctaagaag-′3; 5′-ttcttggcacccacaacctt-′3); *β-actin* (forward 5’tgcctgccatgtatgttgccatcc’3; reverse 5’aacagcaaggtcagacgaaggat’3).

The expression levels of *NPR1*, *TrxH*, and the *β-actin* gene, used for normalisation, were determined as described in [15] by real-time RT-PCR using the GeneAmp 7500 sequence detection system (Applied Biosystems, Foster City, CA, USA). Relative quantification of gene expression was calculated by the Delta-Delta Ct method.

### 4.6. Determination of Soluble K^+^, Ca^2+^, Na^+^, and Cl^−^ Content

The effect of NaCl stress in the presence and absence of MD and Phe on soluble K^+^, Ca^2+^, Na^+^, and Cl^−^ content was determined in leaves and roots of J8-1 seedlings grown under greenhouse conditions. First, leaf and root samples (at least five replicates per treatment) were oven-dried at 65°C and ground to a fine powder. Then, approximately 0.1 g was extracted with milliQ water (1/10 *w*/*v*) at 50 °C for 3 h and shake-incubated for 24 h at 30 °C.

The concentrations of the soluble nutrients analysed were determined by ion-selective electrodes (IonMeter, Nsensors ©) that were previously calibrated with standard solutions of NaCl (for Na^+^ and Cl^−^), CaCl_2_ (for Ca^+2^), and KCl (for K^+^).

### 4.7. Statistical Analysis

The data were analysed by one-way or two-way ANOVA using SPSS 22 software (Chicago, IL, USA). Means were separated with the Duncan’s Multiple Range Test (*p* < 0.05).

## 5. Conclusions

As a general conclusion, in this work we have demonstrated that the plum line J8-1 is tolerant to NaCl in terms of plant growth and plant performance (chlorophyll content and chlorophyll fluorescence parameters, shoot biomass and leaf and root water contents) under the tested conditions. In the presence of NaCl, the MD treatment produced the highest SA and JA increases, but it also induced the expression of *NPR1* and *TrxH* transcripts. These results, similar to those reported by other authors, suggest that the *NPR1*/*TrxH* interaction, along with SA and JA accumulation, may play an important role in the tolerant response of the J8-1 plum line to salt stress. The biosynthetic pathways of SA, JA and ABA take place in the chloroplast [43], and this organelle is rapidly affected by salt stress [2]. Therefore, a connection of the SA, JA, and ABA pathways and qN with the expression of *NPR1* and *TrxH*, mediated by the redox state of the chloroplast can be suggested.

Finally, the results led us to think that the new SA synthesis pathway demonstrated in peach seemed not to be operative in plum under in vitro conditions. However, MD could be involved in the SA biosynthesis under NaCl stress conditions in plum plants under greenhouse conditions. In the transgenic plum line J8-1 a crosstalk between different signalling pathways (NPR1/Trx and SA/JA) leading to salinity tolerance is suggested.

## Figures and Tables

**Table 1:** Effect of salt stress (100 mM NaCl), in the presence or absence of MD and Phe treatments, on total ascorbate (TASC) and reduced ascorbate (ASC) content in micropropagated J8-1 shoots. Data represent the mean ± SE of at least four repetitions. Different letters in the same column indicate significant differences according to Duncan’s test (*p* ≤ 0.05).

	Treatment	TASC (µmol g^−1^ FW)	ASC (µmol g^−1^ FW)	Ascorbate Redox State
−NaCl	Control	1.2 ± 0.16 c	0.9 ± 0.08 ab	0.74 ± 0.03 a
	MD Phe	1.3 ± 0.03 bc 1.7 ± 0.03 c	0.9 ± 0.04 ab 1.2 ± 0.05 a	0.73 ± 0.01 a 0.69 ± 0.02 a
+NaCl	Control	1.7 ± 0.09 ab	1.2 ± 0.03 a	0.69 ± 0.05 a
	MD Phe	1.2 ± 0.06 ab 2.1 ± 0.26 a	0.8 ± 0.04 b 1.2 ± 0.16 a	0.72 ± 0.00 a 0.57 ± 0.02 b

**Table 2:** Effect of salt stress (100 mM NaCl), in the presence or absence of MD and Phe treatments, on total glutathione (TGSH) and reduced glutathione (GSH) content in micropropagated J8-1 shoots. Data represent the mean ± SE of at least four repetitions. Different letters in the same column indicate significant differences according to Duncan’s test (*p* ≤ 0.05).

	Treatment	TGSH (nmol g^−1^ FW)	GSH (nmol g^−1^ FW)	Glutathione Redox State
−NaCl	Control	91.1 ± 4.82 c	86.7 ± 4.25 c	0.95 ± 0.01 ab
	MD Phe	90.1 ± 1.94 c 104.9 ± 7.60 c	86.1 ± 1.47 c 98.3 ± 6.99 bc	0.96 ± 0.00 a 0.93 ± 0.00 ab
+NaCl	Control	104.9 ± 6.66 bc	96.9 ± 6.62 bc	0.92 ± 0.00 b
	MD Phe	140.5 ± 6.40 a 120.5 ± 6.55 ab	131.7 ± 6.38 a 112.0 ± 5.63 ab	0.94 ± 0.00 ab 0.93 ± 0.01 ab

**Table 3:** Effect of salt stress (100 mM NaCl), in the presence or absence of MD and Phe treatments, on ascorbate (ASC) and glutathione (GSH, reduced; GSSG, oxidized) contents in the leaves of J8-1 seedlings. Data represent the mean ± SE of at least four repetitions. Different letters in the same column indicate significant differences according to Duncan’s test (*p* ≤ 0.05).

	Treatment	ASC (µmol g^−1^ FW)	GSH (nmol g^−1^ FW)	GSSG (nmol g^−1^ FW)	Glutathione Redox State
−NaCl	Control	4.6 ± 0.7 b	109.5 ± 1.9 a	11.4 ± 1.1 b	0.91 ± 0.01 a
	MD Phe	3.7 ± 0.2 bc 4.5 ± 0.6 b	99.6 ± 5.2 a 114.5 ± 5.4 a	37.5 ± 1.4 a 30.1 ± 2.8 a	0.72 ± 0.02 c 0.79 ± 0.02 b
+NaCl	Control	3.4 ± 0.4 bc	103.5 ± 7.5 a	13.5 ± 1.0 b	0.88 ± 0.01 a
	MD Phe	7.1 ± 1.5 a 1.8 ± 0.2 c	79.3 ± 8.0 b 114.2 ± 6.9 a	36.3 ± 3.3 a 30.2 ± 2.1 a	0.68 ± 0.02 c 0.79 ± 0.01 b

## Supplementary Materials

Supplementary materials can be found at http://www.mdpi.com/1422-0067/19/11/3519/s1.
