# Supplementary material for: The Salt-Stress Response of the Transgenic Plum Line J8-1 and Its Interaction with the Salicylic Acid Biosynthetic Pathway from Mandelonitrile
Source: Int J Mol Sci. 2018 Nov 8;19(11):3519. doi: 10.3390/ijms19113519 (PMC6274726; doi:10.3390/ijms19113519)
Supplement: Supplementary file 1 [file ijms-19-03519-s001.pdf]

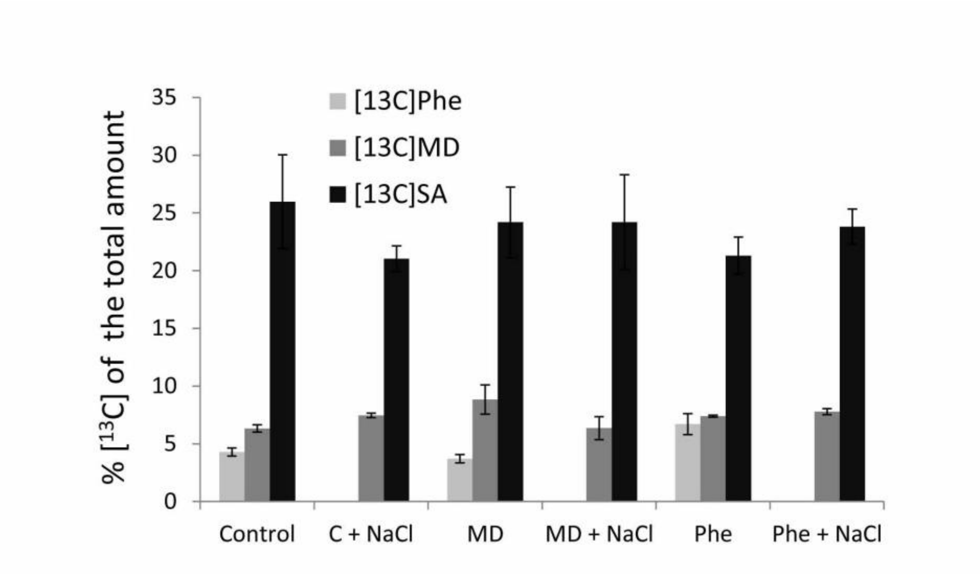

Sup. Figure 1. The % of [13C]Phe, [13C]MD and [13C]SA of the total amount detected in J8-1 micropropagated shoots.

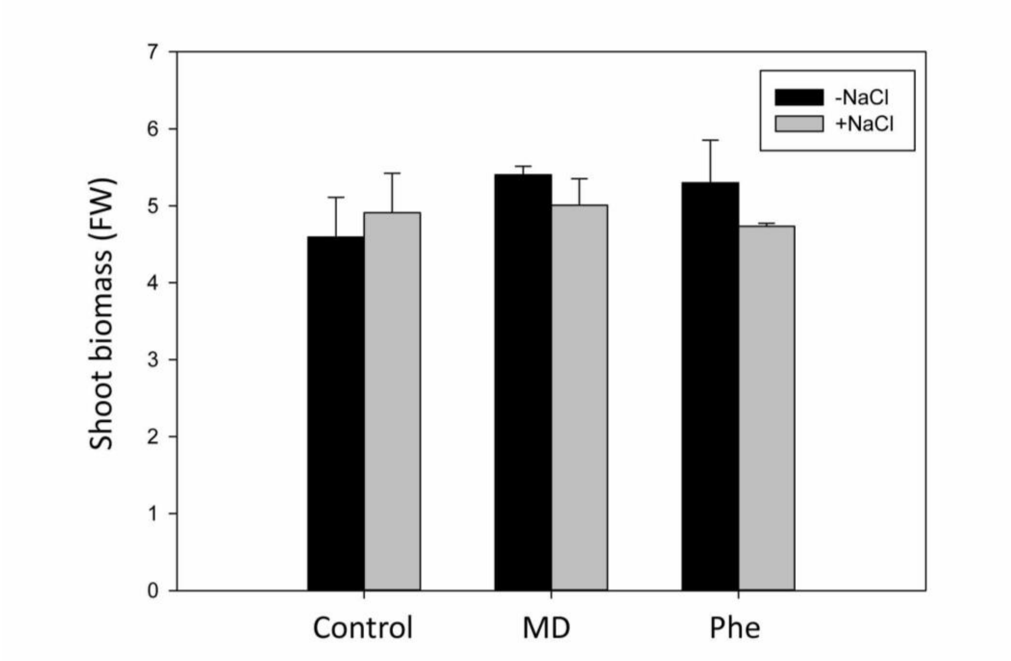

Sup. Figure 2. Effect of NaCl on the shoot biomass of control, MD and Phe treated J8-1 plum seedlings. Data represents the mean ± SE of at least ten repetitions. No statistical differences were observed according to Duncan's test ($p < 0.05$).

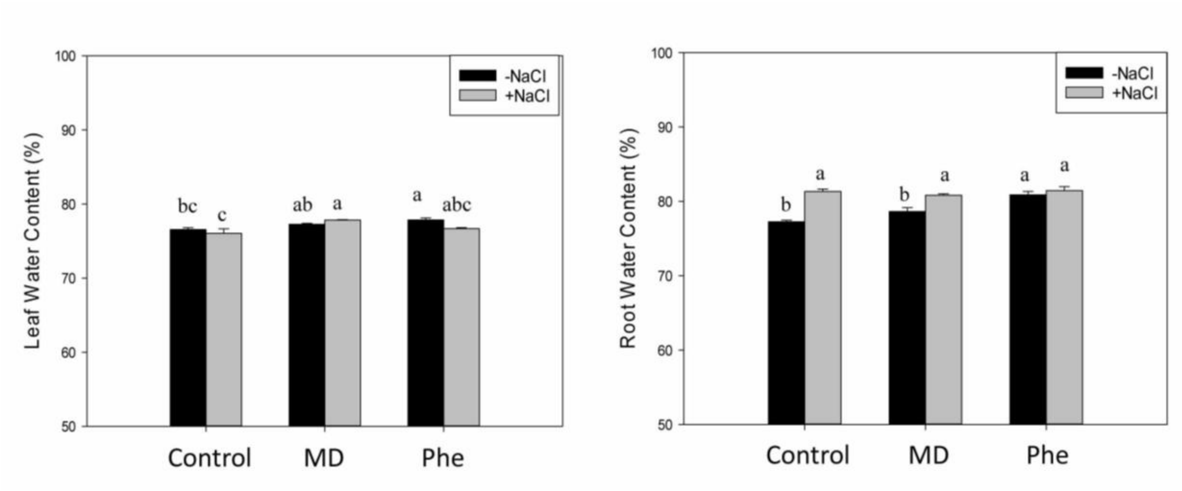

Sup. Figure 3. Effect of NaCl on the water content in the leaves and roots of control and MD- and Phe-treated J8-1 seedlings. Data represents the mean ± SE of at least four repetitions. Different letters indicate statistical significance according to Duncan's test ($p < 0.05$).
